# Platelet-derived- Extracellular Vesicles Promote Hemostasis and Prevent the Development of Hemorrhagic Shock

**DOI:** 10.1038/s41598-019-53724-y

**Published:** 2019-11-27

**Authors:** Ernesto Lopez, Amit K. Srivastava, John Burchfield, Yao-Wei Wang, Jessica C. Cardenas, Padma Priya Togarrati, Byron Miyazawa, Erika Gonzalez, John B. Holcomb, Shibani Pati, Charles E. Wade

**Affiliations:** 1 Center for Translational Injury Research (CeTIR), Department of Surgery, University of Texas Health Science Center at Houston, Houston, McGovern Medical School, Houston, TX USA; 2 Department of Pediatric Surgery, University of Texas Health Science Center at Houston, McGovern Medical School, Houston, TX USA; 3 Vitalant Research Institute, San Francisco, CA USA; 4 Department of Laboratory Medicine, University of California, San Francisco, CA USA

**Keywords:** Platelets, Translational research

## Abstract

Every year more than 500,000 deaths are attributed to trauma worldwide and severe hemorrhage is present in most of them. Transfused platelets have been shown to improve survival in trauma patients, although its mechanism is only partially known. Platelet derived-extracellular vesicles (PEVs) are small vesicles released from platelets upon activation and/or mechanical stimulation and many of the benefits attributed to platelets could be mediated through PEVs. Based on the available literature, we hypothesized that transfusion of human PEVs would promote hemostasis, reduce blood loss and attenuate the progression to hemorrhagic shock following severe trauma. In this study, platelet units from four different donors were centrifuged to separate platelets and PEVs. The pellets were washed to obtain plasma-free platelets to use in the rodent model. The supernatant was subjected to tangential flow filtration for isolation and purification of PEVs. PEVs were assessed by total count and particle size distribution by Nanoparticle Tracking Analysis (NTA) and characterized for cells of origin and expression of EV specific-surface and cytosolic markers by flow cytometry. The coagulation profile from PEVs was assessed by calibrated automated thrombography (CAT) and thromboelastography (TEG). A rat model of uncontrolled hemorrhage was used to compare the therapeutic effects of 8.7 × 10^8^ fresh platelets (FPLT group, n = 8), 7.8 × 10^9^ PEVs (PEV group, n = 8) or Vehicle (Control, n = 16) following severe trauma. The obtained pool of PEVs from 4 donors had a mean size of 101 ± 47 nm and expressed the platelet-specific surface marker CD41 and the EV specific markers CD9, CD61, CD63, CD81 and HSP90. All PEV isolates demonstrated a dose-dependent increase in the rate and amount of thrombin generated and overall clot strength. *In vivo* experiments demonstrated a 24% reduction in abdominal blood loss following liver trauma in the PEVs group when compared with the control group (9.9 ± 0.4 vs. 7.5 ± 0.5 mL, p < 0.001>). The PEV group also exhibited improved outcomes in blood pressure, lactate level, base excess and plasma protein concentration compared to the Control group. Fresh platelets failed to improve these endpoints when compared to Controls. Altogether, these results indicate that human PEVs provide pro-hemostatic support following uncontrolled bleeding. As an additional therapeutic effect, PEVs improve the outcome following severe trauma by maintaining hemodynamic stability and attenuating the development of ischemia, base deficit, and cardiovascular shock.

## Introduction

Traumatic injuries remain a major cause of death worldwide^1^. Uncontrolled bleeding due to trauma and resulting hemorrhagic shock contribute to more than 50,000 potentially preventable deaths per year in the United Sates, and more than 500,000 worldwide with mortality remaining around 14% in the most advanced trauma centers^2,3^. Contributing to uncontrolled bleeding is an intrinsic dysregulation of the blood coagulation system known as trauma-induced coagulopathy (TIC). TIC is associated with poorer outcomes including increased mortality^4^. Apart from rapid control of the source of bleeding, the primary treatment of uncontrolled bleeding and hemorrhagic shock is fluid resuscitation using non-blood or blood components to achieve hemostasis^5^. Although no large-scale clinical studies exist to either support or refute the use of non-blood components for fluid resuscitation, previous studies indicated that aggressive volume replacement, especially due to a large amount of non-blood isotonic fluid, may have negative effects in the setting of uncontrolled bleeding^6^.

Use of blood components is another resuscitative strategy. This strategy recommends transfusion of whole blood, red blood cells (RBCs), plasma and/or platelets (PLTs). The Pragmatic Randomized Optimal Platelet and Plasma Ratios (PROPPR) study, a multicenter clinical trial, reported that higher plasma and platelet ratios early in resuscitation were associated with an improvement in hemostasis and a decrease in hemorrhage-associated mortality in adult trauma patients^7^. Furthermore, transfusion of PLTs alone has been found to ameliorate TIC^8,9^ and improve survival of trauma patients^10–12^.

While the use of PLTs in uncontrolled bleeding raised high expectations in clinical settings, the use of platelet-derived extracellular vesicles (PEVs), a heterogeneous pool of vesicles released from PLTs, may have many advantages over the cell-based approach^13,14^. Short shelf life, reduction in cell function over time, and in some cases, occurrence of transfusion-related acute lung injury are some of the major limitations in the use of PLTs^15–18^. PEVs share many functional features with PLTs such as high procoagulant capacity^19^, but could provide an alternative to PLTs for transfusion given their stability following freeze-thaw cycles^20^. Such characteristics of PEVs effectively remove the logistical barriers of shelf-life limitations, storage, transportation and use in austere environments^14^. Furthermore, our group demonstrated that patients with TIC exhibited a reduction in PEVs levels^19^, indicating the importance of their hemostatic role following injury.

In the present study, we designed a series of *in vitro* and *in vivo* experiments to evaluate the procoagulant effects of PEVs and their ability to treat TIC and improve the outcome of trauma patients. We hypothesized that treatment with human PEVs promote hemostasis, reduce blood loss and attenuate the progression to hemorrhagic shock following severe trauma.

## Materials and Methods

### Preparation of fresh platelets (FPLTs)

Four PLTs units were purchased from the Gulf Coast Regional Blood Center (Houston, Texas). In brief, PLTs were prepared through centrifugation and filtration followed by resuspension in plasma, the preparation is known as platelet-rich plasma method^21^. For our experiments fresh platelets (FPLTs) were used 2 to 5 days after collection. On the day of the experiment, FPLTs were centrifuged and washed 3 times (931 RCF for 20 min at room temperature) in Calcium-free phosphate buffered saline (PBS) containing 0.02 U/ml apyrase and 1.0 µM prostacyclin (PGI2) to inhibit PLT-PLT interactions^22^. FPLTs were counted using an automated blood cell counter (Hemavet 950FS, DrewScientific, Waterbury, CT, USA) on the same day for *in vivo* experiments. The supernatant collected from the first centrifugation was stored at −20 °C for isolation of PEVs. Human platelets were used to increase the translational significance of the study.

### Isolation of PEVs by sequential filtration

The supernatant collected from each PLT unit was thawed and processed to isolate the extracellular vesicles (EVs) using sequential filtration method as previously reported^23^ and in agreement with the recent recommendations by the International Society of Extracellular Vesicles^24^. In brief, the PLTs supernatant was passed through a 0.2 μm membrane to remove any floating cell debris. The supernatant was then loaded into the Millipore LabScale tangential flow filtration (TFF) system equipped with a Biomax 500 kDa Pellicon filter (Millipore, Billerica, MA). Three volume exchanges were performed with 500 mL calcium-free PBS and a target feed pressure below 20 pounds per square inch (psi) and retentate pressure below 10 psi. A final volume reduction step was then performed, with PEVs recovered in a final volume of approximately 10 ml of PBS. The procedure was performed at room temperature and the resultant PEVs concentrate was stored at −20 °C until the day of the experiment.

### Particle size distribution and quantification of PEVs

To determine the particle size distribution and the number of the PEVs, nanoparticle tracking analysis was carried out using Nanoparticle Tracking Analysis (NTA) (NanoSight; alpha nanotech, Raleigh, NC) on samples diluted with PBS^25^. The system focuses a laser beam through a suspension of the particles of interest. These are visualized by light scattering using a conventional optical microscope perpendicularly aligned to the beam axis, which collects light scattered from every particle in the field of view. Three separate 30 second video recordings of all events were collected for further analysis by the nanoparticle tracking analysis software. The Brownian motion of each particle is tracked between frames to calculate its size using the Stokes-Einstein equation.

### Flow cytometric characterization of PEVs

PEVs were analyzed for the phenotypic expression of cell surface markers specific for cell of origin (CD41) and the cell membrane tetraspanins (CD9, CD61, CD63 and CD81) and cytosolic protein HSP90 specific for EVs (all antibodies were purchased from Biolegend, San Diego, CA) using flow cytometry. To remove residual particles and precipitates, CD9, CD41, CD61, CD63 and CD81 antibodies were filtered using the Ultrafree®-MC/Durapore®-PVDF centrifugal filter tubes (Millipore, Hayward, CA). Antibody filtrate was used for staining the PEVs. Subsequently, the PEVs were incubated with antibodies at 4 °C for 30 minutes. Stained PEVs were washed with 200 μL of PBS by transferring them to a 0.22 μm Ultrafree-MC centrifugal filter tubes and were spun at 800 × g for 5 minutes. Biocytex Megamix Plus-SSC reference beads of sizes ranging from 0.16 μm, 0.2 μm, 0.24 μm and 0.5 μm were used to determine the relative sizes of PEVs (Thermo Fisher Scientific, Waltham, MA) **(**Fig. S1**)**. Stained PEVs and reference beads were run on a LSR II benchtop flow cytometer (BD Biosciences, San Jose, CA) and the data were analyzed using FlowJo software (Tree Star, Inc., Ashland, OR, USA).

### Measurement of PEV protein concentration

The protein concentration was measured using a commercially available kit (Bradford assay, Pierce Biochemicals). For the *in vitro* coagulation assays, all 4 samples were normalized to a concentration of 0.1 g/ml. Calcium-free PBS was used as vehicle in both *in vivo* and *in vitro* experiments. The ratio of EVs to protein (particle per mL/mg of protein per ml), as a purity assessment, was calculated as shown in previous validation studies^25^.

### Calibrated automated thrombography (CAT) assay

Adapted from previous studies^26–28^, we used CAT assay to measure the overall potential of PEVs to generate thrombin using a calibrated automated thrombogran (CAT; Thrombinoscope, Maastricht, Netherlands). In brief, 10 µl of PEVs, tissue factor (TF) or vehicle under normalized protein concentration were mixed with 70 µl (1:8 ratio) of rat platelet poor plasma. Then, 20 µl of phospholipid 20 µM (MP-Reagent; Thrombinoscope, Maastricht, Netherlands) were added to maximize the sensitivity to TF. Finally, 20 µl of Calcium Chloride (FluCa; Thrombinoscope, Maastricht, Netherlands) were added to initiate the reaction. The variables analyzed were lag time (min), endogenous thrombin potential (ETP, nM/min), peak height (peak, nM), time to peak (ttpeak, min) and rate calculated as peak/ttpeak - lag time (nM/min). The variables were calculated by Thrombinoscope software (Fluoroskan Ascent, Thrombinoscope 5.0, Diagnostica Stago, Parsippany, NJ).

### Viscoelastic hemostatic assay

The characteristics of the clot were assessed by thromboelastography (TEG) (TEG 5000 Thromboelastograph Analyzer, Haemoscope Corporation, Niles, IL) according to the manufacturer’s recommendations. The assay was performed using citrated rat plasma (thawed) and citrated rat whole blood (fresh).

Plasma: 875 µl of rat platelet poor plasma was mixed with 125 µl of PEVs, TF, or Vehicle (1:8 ratio) followed by exposure to CaCl_2_ and kaolin to start the coagulation reaction.

Whole blood: By measuring the hematocrit we adjusted the proportions of blood and PEVs sample to keep the same proportions (1:8 ratio) used with plasma samples. A volume of 927 µl of fresh blood was mixed with 72.5 µl of PEVs or vehicle followed by exposure to CaCl_2_ and kaolin to start the coagulation reaction. The variables analyzed were the α-angle (degrees) and the maximum amplitude (MA, mm). As suggested^29^, changes in TEG were compared to the parameters obtained from the same investigated animals and not from human standards.

### Rat model of uncontrolled bleeding

The experimental procedures were approved by the Institutional Animal Care and Use Committee of the University of Texas Health Science Center at Houston. Animals were handled and maintained in accordance with the Guide for the Care and Use of Laboratory Animals. Following at least 3 days of acclimatization and *ad libitum* access to water and food, 32 male Sprague-Daley rats (340.5 ± 20 grams body weight) were anesthetized and surgically prepared as previously described^30^. In brief, under anesthesia, using 1–3% isoflurane titrated to allow systemic anesthesia under spontaneous breathing and buprenorphine for additional pain control, the animals were surgically prepared to cannulate the femoral artery and the jugular vein. A 3 French polyurethane catheter (Norfolk Access, Skokie, Illinois) was washed with heparinized saline (15 U/ml) to avoid intra-catheter blood clotting. Following the surgical procedure and without interrupting anesthesia, isoflurane was titrated to achieve a mean arterial pressure (MAP) between 90 and 110 mmHg, allowing collection of arterial blood for baseline assessment. Following the collection of baseline readings, a midline laparotomy was performed and approximately 50% of the middle hepatic lobe was excised and PEVs, FPLTs or Vehicle were infused after 60 seconds. The degree of injury was monitored by collecting the weight of the excised liver and measuring the bleeding surface area. Abdominal bleeding after liver injury was quantified with pre-weighed gauze pads measured every 15 minutes during 60 minutes. At 60 minutes post-injury, another arterial blood sample was collected for biochemical analysis followed by euthanasia.

### Grouping and experimental design

The animals were divided in three experimental groups: (i) control group (n = 16; received 5 ml of PBS in the jugular vein catheter); (ii) FPLT group (n = 8; received 8.7 × 10^8^ FPLTs resuspended in 3 ml of PBS + 2 ml of PBS to flush the line); and (iii) PEV group (n = 8; received 7.8 × 10^9^ PEVs resuspended in 3 ml of PBS + 2 ml of PBS to flush the line). The experiments were done in pairs on the same day and were randomly assigned to either of the experimental groups. Each treatment animal was paired with a control animal. FPLT group was treated with washed platelets from one of the donors shown in Table 1A. The concentration of PEVs used was adapted from the maximal effective dose utilized in the calibrated automated thrombography assay reported here and the concentration of FPLT was adapted from a previous study^22^. PEVs group was treated with a pool of the 4 donors normalized to a protein concentration of 1.0 × 10^5^ µg/ml. Treatments were given intravenous via the jugular vein catheter 60 seconds after the excision of the liver.

**Table 1:** (A) Age (years), gender, platelet count (PLT/µL) and volume (mL) from each unit along with (B) their corresponding PEV isolate prepared from the supernatant of each platelet unit. Protein concentration presented as mg/mL, EVs count as EVs/mL, particle:protein ratio as EVs/mg and Size as mean ± SEM and mode.

(A) Platelet Unit Information					(B) Corresponding PEV Isolate				
Donor	Age	Gender	PLT count	mL	mg/mL	EVs/mL	EVs/mg	Size (nm)	
1	32	Female	846,000	57	113.0	4.94 × 10^11^	4.4 × 10^8^	176 ± 66	134
2	21	Male	775,500	34	120.7	2.38 × 10^11^	2.0 × 10^8^	168 ± 83	121
3	58	Female	900,000	60	173.3	3.42 × 10^11^	2.0 × 10^8^	115 ± 49	96
4	19	Male	1,026,000	66	105.2	3.42 × 10^11^	4.5 × 10^8^	155 ± 63	107

### Blood physiological and biochemical assessment

Blood pressure was measured using a hemodynamic monitor (Power Lab, AD Instruments, Colorado Springs, CO) connected to the femoral artery. Blood levels of oxygen (O_2_), carbon dioxide (CO_2_), bicarbonate (HCO_3_), base excess (BE) and pH were quantified by sampling and measuring 200 μl of arterial blood with a blood gas analyzer (Stat Profile Critical Care Xpress, NOVA Biomedical, Waltham, WA) and lactate using a handheld testing system (Stat Strip Xpress Lactate Hospital Meter, NOVA Biomedical, Waltham, WA). The manual microhematocrit method was used to determine the hematocrit (Hct) and a refractometer for the plasma protein concentration.

### Organs wet to dry (W/D) ratio

To determine water content in heart, lung, kidney, liver and intestine, samples of fresh organs were collected and weighed immediately after euthanasia. The organs were then placed in a 60 °C vacuum oven and weighed again 72 hours later after water evaporation.

### Statistical analysis

The results were analyzed using GraphPad Prism 8 (GraphPad Prism version 8.1, GraphPad Software, La Jolla, CA). Outcome data collected at a single time point was compared using One-way analysis of variance (ANOVA) or Kruskal-Wallis test when normality was not met. Data collected from multiple time points were analyzed using two-way analysis of variance (2-way ANOVA) with Tukey post hoc test to compare the differences among groups at each time point. All values in tables are expressed as mean ± standard error of the mean (SEM). Differences were considered significant when the p-value was equal or smaller than 0.05.

## Results

### Characterization of PEVs

**Figure 1:**
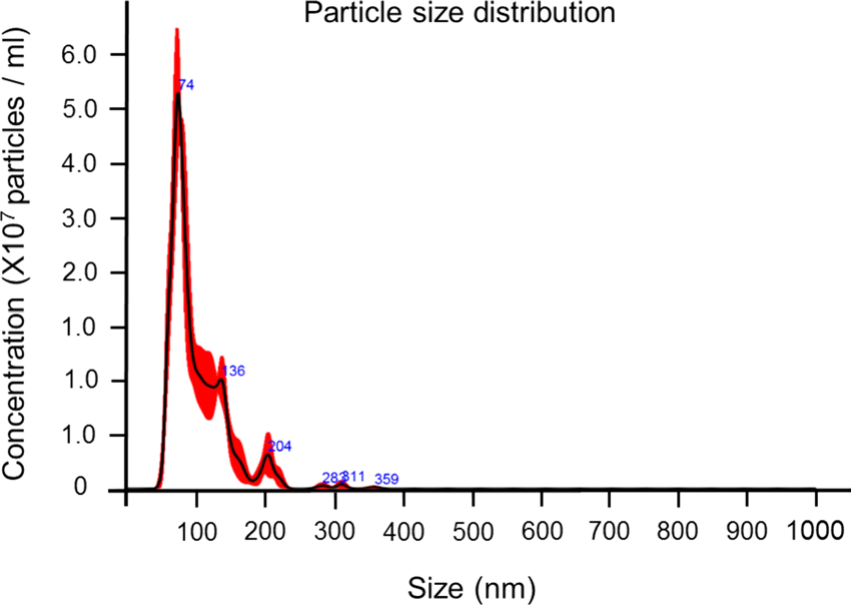
Averaged PEVs Concentration and Size from a pool of EVs from the 4 donors recorded by Nanoparticle Tracking Analysis. Highest pick represents the mode. Error bars in red indicate ± SEM.

The PEVs obtained from four different donors showed a similar profile based on size distribution and surface markers. The age and gender of the donors as well as the platelet counts from each unit are presented in Table 1A. The protein concentration, EV count and size assessment (mean and mode) were determined and are presented in Table 1A and Fig. 1. It was observed that all the PEVs obtained from four different donors expressed platelet-specific marker CD41 **(**Figs. S2 and S3**)**. Additionally, they also expressed tetraspanin proteins CD9, CD61, CD63, CD81 and cytosolic protein HSP90 that are known to be present on EVs **(**Figs. S2 and S3**)**. Variation in the expression of EV-specific tetraspanin proteins in our study in comparison to the other studies might be attributed to the different technical procedures followed to isolate EVs^23,31,32^. Likewise, the use to 0.2 µm filter to remove cell derbies, for the pure and highly efficient isolation of EVs from a platelet unit exhibited a highly pure homogeneous EV population. Although, the final isolated EV preparation may not represent the entire EV population from a platelet unit.

### Procoagulant effect PEVs *in vitro*

**Figure 2:**
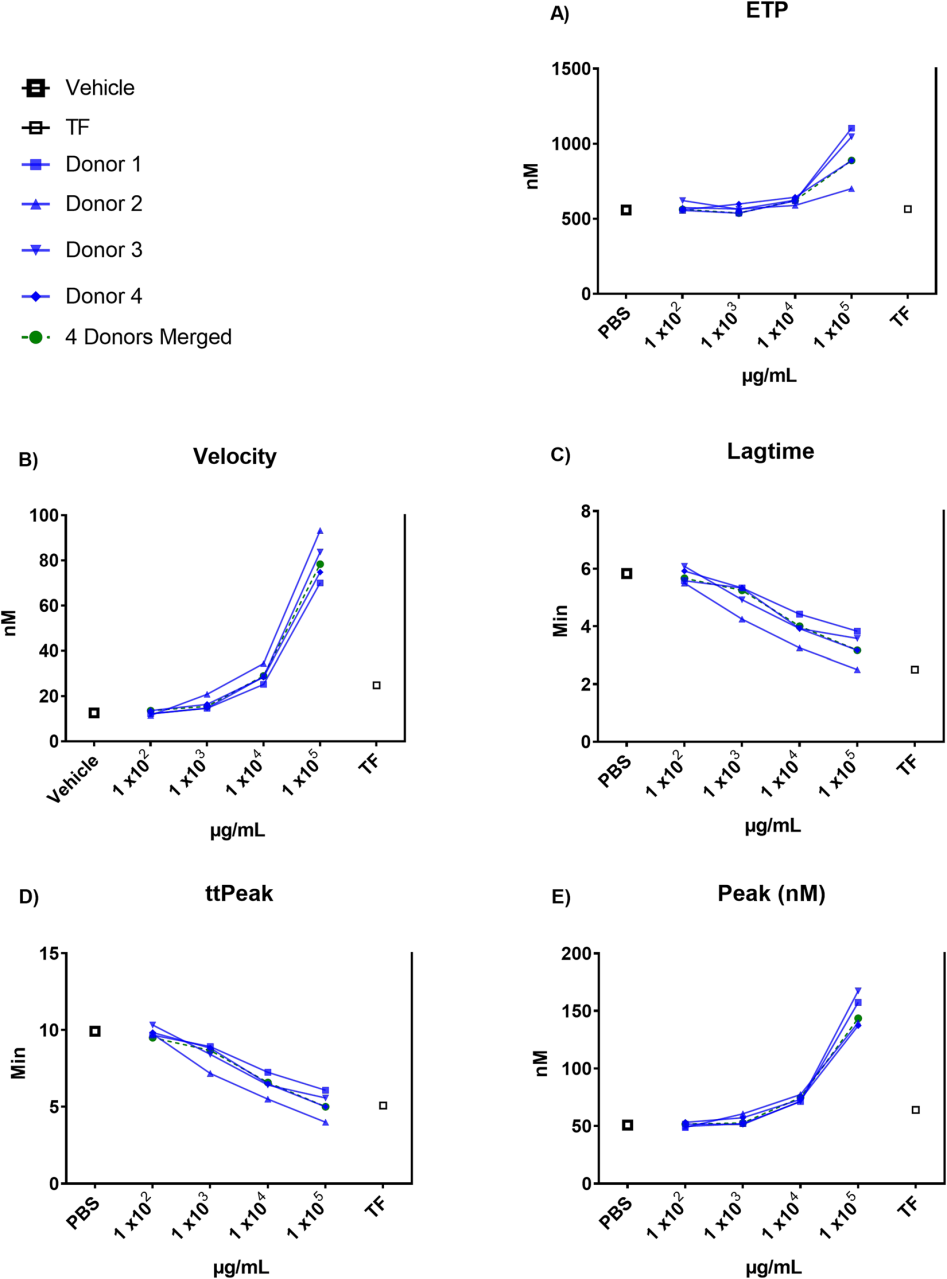
Dose-response procoagulant effect of PEVs. Multiple dilutions of PEVs isolates from 4 different donors alone and combined were exposed to rat fresh frozen plasma. Generation of thrombin was assessed by CAT. Rh-TF and PBS were used as positive and negative control respectively. ETP = endogenous thrombin potential, ttPeak = time to peak, TF = tissue factor.

We tested the procoagulant capabilities of each of the isolated PEVs samples both separately and as a pool using two different methods (Fig. 2 and Table 2). Thrombin generation assay in rat plasma shows that, compared to vehicle, PEVs isolated from each of the 4 donors exhibited a dose-response effect increasing the rate of the reaction as shown by reductions in lagtime and ttPeak and increased velocity. TF, used as positive control, exhibited a strong effect on lagtime and ttPeak similar to the highest dose of PEVs, but only a mild effect on velocity. Likewise, PEVs exhibited a dose-dependent effect on Peak and ETP, suggesting a contribution to the overall amount of thrombin generated. **(**Fig. 2**)**. Thromboelastography analysis was performed in rat plasma and whole blood using the highest dose PEVs (3.2 × 10^8^ EVs) shown in Fig. 2 and the same proportions of plasma (1:8 ratio). In whole blood, PEVs treatment demonstrated an increase in MA compared to Vehicle and an increase in the α-angle parameter. In plasma PEVs treatment also demonstrated an increase in MA compared to Vehicle but no difference was observed in the α-angle **(**Table 2**)**. The changes in MA and the α-angle indicates that the overall strength of the clot generated is greater in the presence of PEVs.

**Table 2:** Coagulation profile of PEVs assessed by Thromboelastography.

Group	Plasma		Whole Blood	
	α-angle (degrees)	MA (mm)	α-angle (degrees)	MA (mm)
PEVs, n = 4	81.6 ± 0.4	36.6 ± 0.5	80.6 ± 0.4	69.9 ± 0.9
Vehicle, n = 4	80.9 ± 0.5	31.6 ± 1	78.8 ± 0.3	65.6 ± 0.9
p value	0.26	0.01*	0.02*	0.02*

PEVs isolation from 4 different donors were exposed to rat fresh frozen plasma. PBS was used as negative control. Difference between mean values were analyzed by student t test, *p < 0.05 vs. Vehicle. Data reported as mean ± SEM. MA = maximum amplitude, PEVs = Platelet-derived extracellular vesicles.

### Blood and hemodynamic parameters from *in vivo* model of uncontrolled bleeding

**Figure 3:**
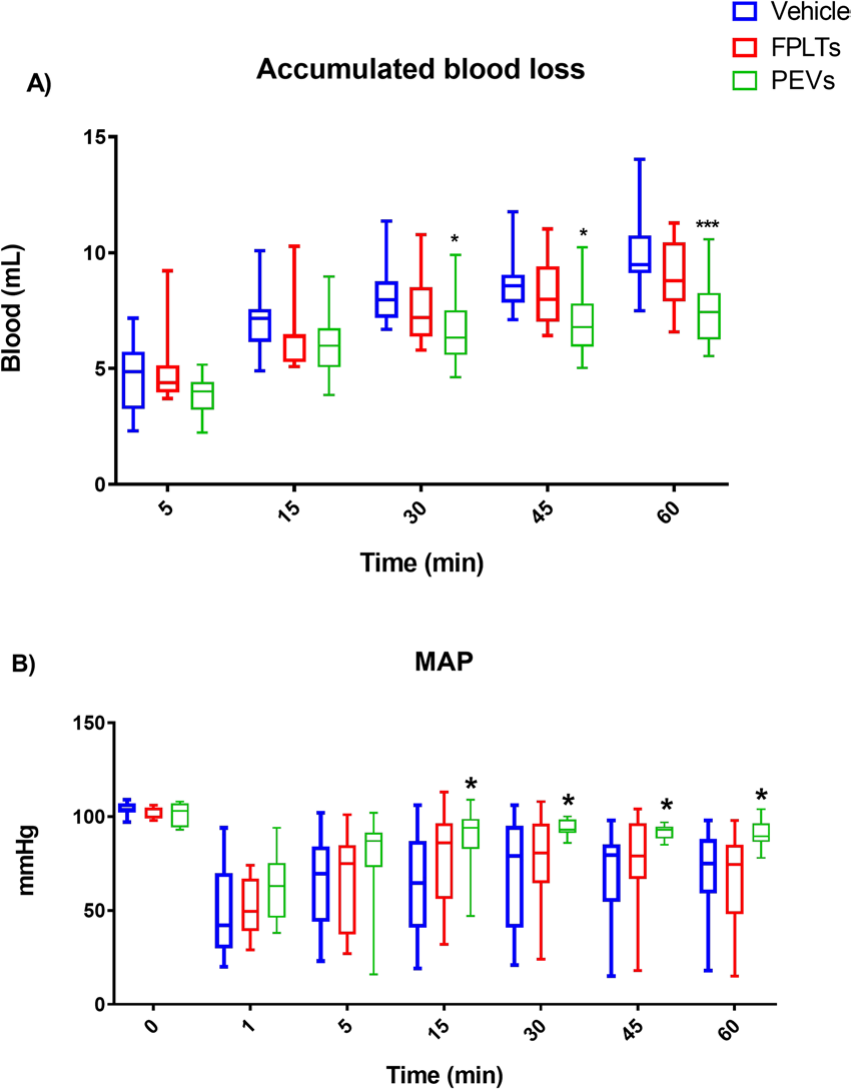
Treatment effect on hemodynamics and hemostasis. (**A**) Bleed volume continuously collected from the peritoneal cavity following liver excision. (**B**) MAP measured from a femoral artery catheter showing a rise in blood pressure in PEVs treated group. Vehicle, n = 16; fresh platelets (FPLTs), n = 8; platelet extracellular vesicles (PEVs), n = 8. Box plots Showing the median ± 99 percentile and compared by 2-way ANOVA, *p < 0.05 vs. Control. ***p < 0.001 vs. Control.

The body weight and blood biochemical parameters at baseline (hematocrit, plasma, lactate, BE, CO_2_, O_2_ and pH) were comparable among the Vehicle, FPLT and PEVs groups **(**Table 3A**)**. The degree of the injury was similar in all groups, as shown by the weight of the liver excised, the bleeding surface and the MAP 60 seconds after liver excision **(**Table 3B**)**. Assessment of peritoneal bleed during the first 30 minutes postinjury demonstrated an accumulation of 8.1 mL of blood on average in the control group and 7.6 mL in the FPLTs group vs. a significant reduction to 6.6 mL in accumulated blood in the PEVs group. The difference in bleeding between control and PEVs groups was the greatest at 60 minutes postinjury (9.9 and 7.5 mL, respectively) **(**Fig. 3A**)**. Likewise, mean MAP was similar among groups at baseline **(**Table 3A**)** and dropped to 64, 70, 68, and 67.1 mmHg at 15, 30, 45 and 60 min postinjury in the control group compared to 88.5, 93.6, 92, and 90.9 mmHg in the PEVs indicating a significant improvement with PEVs therapy. In contrast, FPLT infusion failed to improve the MAP vs. Vehicle **(**Fig. 3B**)**. Arterial blood sampling collected 60 minutes postinjury demonstrated that the Htc, O^2^, CO^2^ and pH had a similar change in all groups **(**Table 4**)**. However, the total plasma protein dropped to 3 g/dL in the Vehicle and FPLTs groups compared to a 4 g/dL in the PEVs group suggesting a significant improvement in oncotic pressure. Lactate and BE were clearly increased in Vehicle (5 mmol/L and −2.9 mmol/L, respectively) and FPLTs groups (3 mmol/L and −5.6 mmol/L) compared to maintained levels in the PEVs group (1 mmol/L and 3.5 mmol/L) indicating a mitigated metabolic decompensation in this group. Lung, heart, kidney and liver water content, assessed by WD ratio, was similar among the treatment groups and untreated controls **(**Table 4**)**.

**Table 3:** Comparisons among groups at baseline and assessment of injury severity.

	Vehicle, n = 16	FPLT, n = 8		PEVs, n = 8		ANOVA	
	Mean	SEM	Mean	SEM	Mean	SEM	p value
**(A) Baseline values**							
Body weight (g)	338±	5	351±	8	334±	3	0.17
Blood volume (mL)	22±	0.3	23±	0.5	22±	0.2	0.17
MAP (mmHg)	104±	1	103±	1	101±	2	0.33
Hematocrit (%)	42.7±	0.8	44.5±	1.4	44.6±	1.4	0.33
Plasma protein(g/dL)	4.6±	0.1	5.3±	0.3	4.7±	0.2	0.09
Lactate (mmol/L)	0.6±	0.0	0.6±	0.1	0.6±	0.1	0.96
BE (mmol/L)	5.3±	0.7	2.7±	1.0	6.3±	0.8	0.08
CO^2^ (mmHg)	47±	3	46±	3	44±	1	0.10
O^2^ (mmHg)	509±	28	474±	38	499±	33	0.80
pH	7.4±	0.0	7.4±	0.0	7.4±	0.0	0.18
**(B) Injury Severity parameters**							
Excised Liver (g)	1.2±	0.1	1.1±	0.0	1.1±	0.1	0.31
Bleeding Surface (cm2)	1.9±	0.1	1.7±	0.1	1.9±	0.1	0.12
MAP-1 min (mmHg)	51±	6	52±	6	63±	6.4	0.39

(A) BW, hemodynamics and blood gasses were comparable in all groups. (B) The weight, bleeding surface and MAP one minute after liver excision demonstrates a comparable injury among the groups. Data reported as mean ± SEM and compared by 1-way ANOVA.

**Table 4:** Treatment effects on blood parameters and tissue water content.

	Vehicle, n = 16	FPLT, n = 8		PEVs, n = 8		ANOVA	
	Mean	SEM	Mean	SEM	Mean	SEM	p value
Hematocrit (%)	31±	1	29±	1	29±	2	0.47
Plasma protein (g/dL)	3±	0.1	3±	0.1	4±	0.1	0.048*
Lactate (mmol/L)	5±	1	3±	1	1±	0	0.002*
BE (mmol/L)	−2.9±	2.3	−5.6±	2.7	3.5±	0.8	0.013*
CO^2^ (mmHg)	46±	5	45±	5	56±	10	0.52
O^2^ (mmHg)	337±	51	341±	97	515±	68	0.12
Lung (Index)	3.9±	0.17	3.9±	0.04	3.9±	0.04	0.95
Heart (Index)	3.4±	0.08	3.3±	0.03	3.3±	0.05	0.55
Liver (Index)	2.4±	0.04	2.4±	0.06	2.2±	0.03	0.07
Kidney (Index)	2.8±	0.09	2.6±	0.10	2.8±	0.10	0.36

Gasometry and protein concentration measured from blood following 60 min of injury and treatment. Lung, heart, liver and kidney water content was determined by W/D index. Data reported as mean ± SEM and compared by 1-way ANOVA, * p < 0.05 vs. Control.

## Discussion

In the present study, we showed that a well-characterized EV preparation isolated from platelet units have a potent procoagulant effect demonstrated by both *in vivo* and *in vitro* models. Coagulation assays demonstrated that our preparation has a consistent effect, as shown by the similar effects from each of the 4 isolates on both thrombin generation and TEG assays. An important aspect from the results is the comparison between PEVs and TF treatments since a number of studies have suggested that the procoagulant features of PEVs are primarily result from their high TF and phosphatidylserine (PS)^33,34^. This concept is in agreement with our observations given that PEVs dose-dependently increased both the rate and overall amount of thrombin generation. Furthermore, viscoelastic assay demonstrates that in whole blood, PEVs increased the fibrin polymerization rate (α-angle) and the strength of the clot (MA), likely as a result from the interaction with the platelets present in blood. However, the experiment in platelet-free plasma also showed that the strength of the clot was superior in the presence of PEVs suggesting a significant interaction with other components of the clot. A potential explanation for this effect could be the expression of specific integrins in PEVs capable of binding fibrinogen and promoting clot formation^35^, or by a change in fibrin clot structure triggered by the increase in thrombin concentration^36^.

We used the most efficacious dose of PEVs shown by *in vitro* assays to test the therapeutic effect of such treatment on an *in vivo* uncontrolled bleeding model. The experiment demonstrated that PEVs therapy provided an effective hemostatic effect as compared to vehicle-treated animals and superior to FPLTs treated animals. Although a number of publications have suggested that EVs could be employed as a procoagulant drug^37^, the present study is the first to demonstrate the concept in a clinically relevant animal model.

Another important finding from the animal experiments was that PEVs treatment markedly improved the blood pressure, the levels of total protein and lactate in plasma, and the base deficit. The evidence provided cannot explain the order of such outcomes, though, we speculate that, as shown by the MAP and plasma protein concentration, the hydrostatic and oncotic pressure were maintained in the PEVs group as a result of the preserved blood volume. Consequently, perfusion improved in the PEVs group compared to FPLT and Vehicle counterparts, attenuating ischemia and reducing the generation of lactate and preventing the development of metabolic acidosis and cardiovascular shock. The group treated with FPLTs failed to improve the hemodynamics, plasma protein concentration, lactate levels and the base deficit, and although transfused PLTs have shown to improve the outcome in trauma patients^12^, resuscitation with platelets alone in the absence of plasma likely reduces the effectiveness. Likewise, our PEVs preparation is noticeably superior to platelet transfusion from a storage perspective, since platelets can only be used within 5 days post-withdraw and cannot be frozen.

These results are in agreement with previous results from our group demonstrating the decrease in PEVs associated with both TIC^19^ and with a decrease in survival of trauma patients^38^ and with Windelov *et al*. showing that trauma hypocoagulable patients have lower levels of PEVs^39^. Remarkably, Windelov *et al*. found as well that lactate levels and injury severity were inversely correlated with the concentration of PEVs in trauma patients, suggesting the potential role of PEVs to mitigate trauma severity^39^. Moreover, acidosis prevents the release of EVs from platelets, therefore in the present study PEVs administration could be compensating for the loss of endogenous EVs^40^.

Although in our study we did not evaluate the direct effect of PEVs on the endothelium, Miyazawa *et al*. demonstrated that PEVs protects the microvasculature from permeability disrupting factors^41^. Likewise, Mause *et al*. demonstrated that PEVs promote vascular integrity as well as endothelial recovery^42^; therefore, the attenuated decreased in plasma protein concentration shown by the animal experiments is likely a consequence of a PEVs-induced anti-leak effect. Severe sepsis shares a number of features with trauma, and high levels of PEVs have shown to be associated with improved outcomes in septic patients, specifically with lower incidence of multi-organ failure^43^.

To acquire a deep understanding of hemodynamic physiology it is necessary to investigate plasma proteins, the blood cells and the endothelium. In this regard, an important limitation from the *in vitro* coagulation studies is that, our assays did not account for interactions with the endothelium, although the animal experiment partially addressed this limitation. On a different context, a limitation from *in vivo* experiments was the fact that we did not collect blood from the animals in shorter time points, as this approach would have allowed us to explore the dynamics of the biomarkers measured and it would give us the possibility to measure the immediate coagulation effect caused by the treatment. However, this would have affected the assessment of uncontrolled bleeding and the parallel hemodynamic compensatory response.

## Conclusion

The present findings indicate that the therapeutic benefits of platelets during hemorrhage is partly due to the effects of platelet-derived EVs. Here we employed a PEVs preparation showing an efficacious hemostatic effect and the advantage of frozen storage. Furthermore, the results indicate that as an additional therapeutic effect, PEVs improve the outcome following severe trauma by maintaining hemodynamic stability and mitigating the development of ischemia and metabolic acidosis. The study is the first to demonstrate evidence to use PEVs as an alternative therapy providing hemostasis for trauma patients. Before these promising findings can be translated into clinical practice, further research is needed to expand the characterization of the PEVs isolates with a particular emphasis to elucidate donor-to-donor variability within a large selection of donors.

## Supplementary information

Supplemental Material (Figure S1 and S2)
