# Supplementary material for: Platelet-derived- Extracellular Vesicles Promote Hemostasis and Prevent the Development of Hemorrhagic Shock
Source: Sci Rep. 2019 Nov 27;9:17676. doi: 10.1038/s41598-019-53724-y (PMC6881357; doi:10.1038/s41598-019-53724-y)
Supplement: Supplementary file 1 — Supplemental Material (Figure S1 and S2) [file 41598_2019_53724_MOESM1_ESM.pdf]

Platelet-derived- Extracellular Vesicles Promote Hemostasis and Prevent the Development of Hemorrhagic Shock

Ernesto Lopez1; §, Amit K. Srivastava2, §, John Burchfield1, Yao-Wei Wang1, Jessica C. Cardenas1, Padma Priya Togarrati3, Byron Miyazawa4, Erika Gonzalez1, John B. Holcomb1, Shibani Pati4, Charles E. Wade1

1Center for Translational Injury Research (CeTIR), Department of Surgery, University of Texas Health Science Center at Houston, Houston, McGovern Medical School, Houston, TX, USA;

2Department of Pediatric Surgery, University of Texas Health Science Center at Houston, McGovern Medical School, Houston, TX, USA; 3 Vitalant Research Institute, San Francisco, CA, USA; 4Department of Laboratory Medicine, University of California, San Francisco, CA, USA.

Corresponding Author:
Ernesto Lopez, MD., Ph.D.,
Center for Translational Injury Research (CeTIR),
Department of Surgery, McGovern Medical School,
University of Texas Health Science Center at Houston,
6431 Fannin Street, Houston, Texas, USA
Phone: 713-500-7439
Fax: 713-500-0685
Email: jose.e.lopezvalencia@uth.tmc.edu

[§]*EL and AKS contributed equally to this work.*

Supplemental Figures:

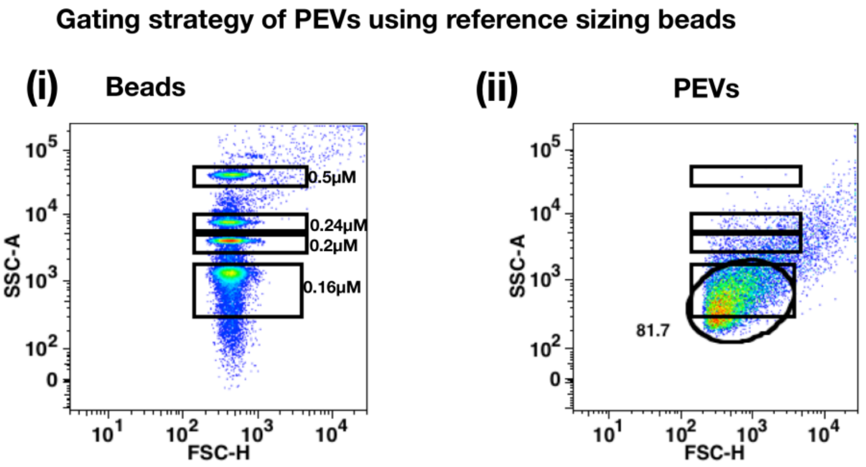

Figure S1: *(i) Biparametric SSC-A vs. FSC-H plot showing location of the 0.16 µm, 0.2 µm, 0.24 µm and 0.5 µm sized Megamix Plus-SSC beads (ii) determination of the strategy to gate PEVs in relation to the Megamix Plus-SSC beads.*

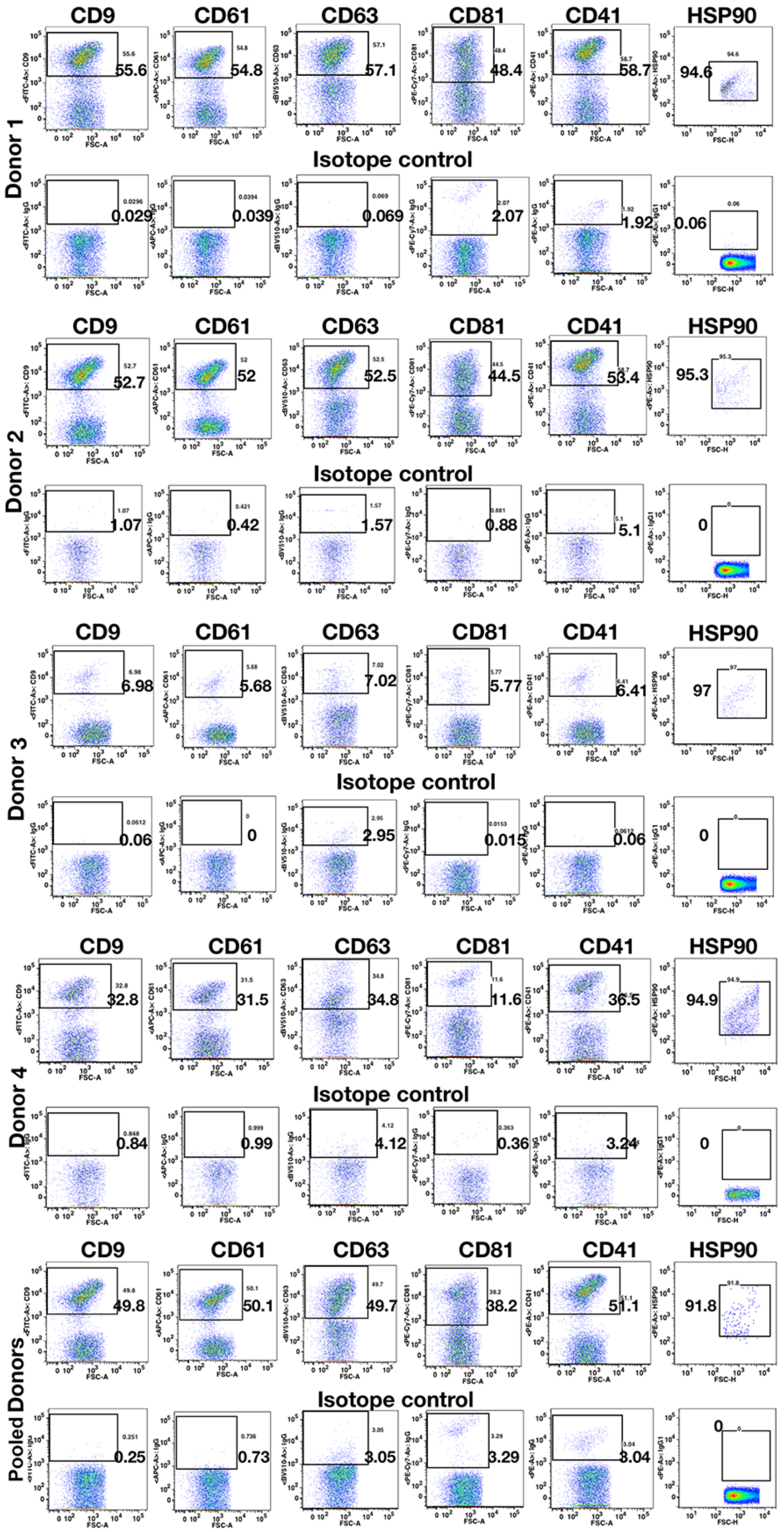

Figure S2: *Flow cytometric characterization of expression of tetraspanin markers, CD9, CD61, CD63, CD81, cytosolic protein HSP90 and platelet-specific marker CD41 on PEVs derived from donors 1, 2, 3 and 4, as well as the pool of all 4 donors merged.*
